# Transcatheter embolization for duodenal ulcer bleeding originating from cystic artery erosion

**DOI:** 10.1186/s42155-024-00470-6

**Published:** 2024-07-20

**Authors:** Peng Hu, Guangwen Chen, Jingpeng Wei, Rengui Huang, Yaochang Luo

**Affiliations:** 1 Department of Interventional Radiology, The First Affiliated Hospital of Guangxi University of Chinese Medicine, Nan Ning, China; 2 Department of Gastroenterology, The First Affiliated Hospital of Guangxi University of Chinese Medicine, Nan Ning, China

**Keywords:** Duodenal ulcer bleeding, Cystic artery, Transcatheter embolization, Upper gastrointestinal bleeding

## Abstract

**Background:**

Ulcer erosion into the cystic artery is a rare cause of bleeding in duodenal ulcers, with only a limited number of cases described in the literature. Historically, treatment has predominantly involved surgical intervention. We present three cases of duodenal ulcer bleeding due to cystic artery erosion, which were successfully managed with cystic artery embolization.

**Case presentation:**

This case series includes three male patients with duodenal ulcer bleeding, aged 90, 81, and 82 years, respectively, and no prior history of biliary system disorders. The ulcer locations were identified as two in the post-bulbar region and one in the anterior bulb. After the failure of medical and endoscopic treatment, transcatheter arterial embolization was adopted. Initial angiography did not reveal any contrast medium extravasation. Empirical embolization of the gastroduodenal artery using gelatin sponge particles and coils failed to achieve hemostasis. Super-selective cystic artery angiography confirmed the source of bleeding as the cystic artery. One patient was embolized with gelatin sponge particles and coils, while the other two patients were embolized with N-butyl-cyanoacrylate. All patients achieved successful hemostasis without gallbladder infraction.

**Conclusions:**

Cystic artery embolization proved to be a minimally invasive technique for achieving hemostasis in these cases, indicating that it may be a safe and effective alternative to surgery for this uncommon cause of upper gastrointestinal bleeding. Validation through further studies is warranted.

## Background

Duodenal ulcer bleeding is the most common cause of non-variceal upper gastrointestinal bleeding (UGIB), often originating from the gastroduodenal artery and pancreaticoduodenal arcades. Erosion of the duodenal ulcer into the cystic artery is rare, a few cases have been reported [1–9]. Most treatment options have historically involved surgical procedures, however direct cystic artery embolization has been rare utilized. In this study, we present 3 cases of duodenal ulcer hemorrhage resulting from erosion into the cystic artery and elaborate on the employment of cystic artery embolization for their management.

## Case presentation

### Case1

**Fig. 1:**
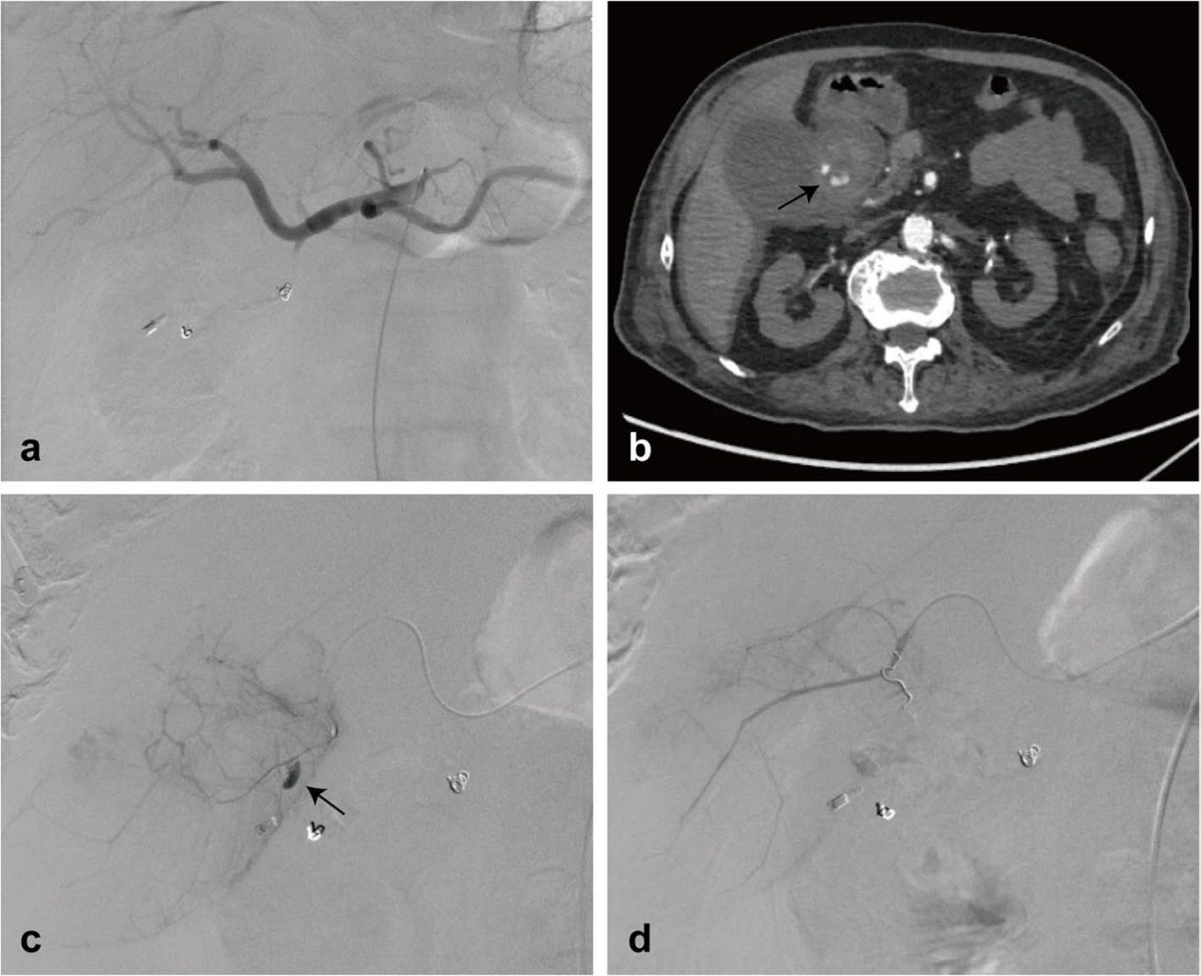
Case 1. **a** Angiography after empirical gastroduodenal artery embolization shows no positive sign. **b** Contrast-enhanced computed tomography demonstrates contrast extravasation near the gallbladder (arrow). **c** Super-selective angiography of the cystic artery demonstrates contrast extravasation (arrow). **d** Post-embolization angiography confirms successful occlusion of the bleeding branches of the cystic artery with gelatin sponge particles and coils

A 90-year-old male patient was admitted with type II respiratory failure due to chronic obstructive pulmonary disease. He had a history of coronary stent implantation and was on daily antiplatelet treatment with aspirin, with no history of biliary system disease. Two weeks after admission, the patient developed melena, which progressed to massive hematemesis and hematochezia, requiring transfusion and vasoactive therapy. Emergency endoscopy identified a post-bulbar duodenal ulcer with active bleeding, which was unresponsive to endoscopic hemostasis. Subsequently, the patient underwent emergency angiography, which did not identify any positive signs, and empirical gastroduodenal artery embolization was performed using gelatin sponge particles and coils (Fig. 1a). However, the patient remained hemodynamically unstable after the procedure. Additional computed tomography (CT) imaging revealed contrast extravasation in close proximity to the gallbladder (Fig. 1b). Super-selective catheterization of the cystic artery was performed, revealing contrast extravasation (Fig. 1c). The bleeding branches of the cystic artery were embolized with gelatin sponge particles and coils (Fig. 1d). After the embolization, the patient remained hemodynamically stable for four months without any recurrent gastrointestinal bleeding. Ultimately, the patient died due to respiratory failure.

### Case2

**Fig. 2:**
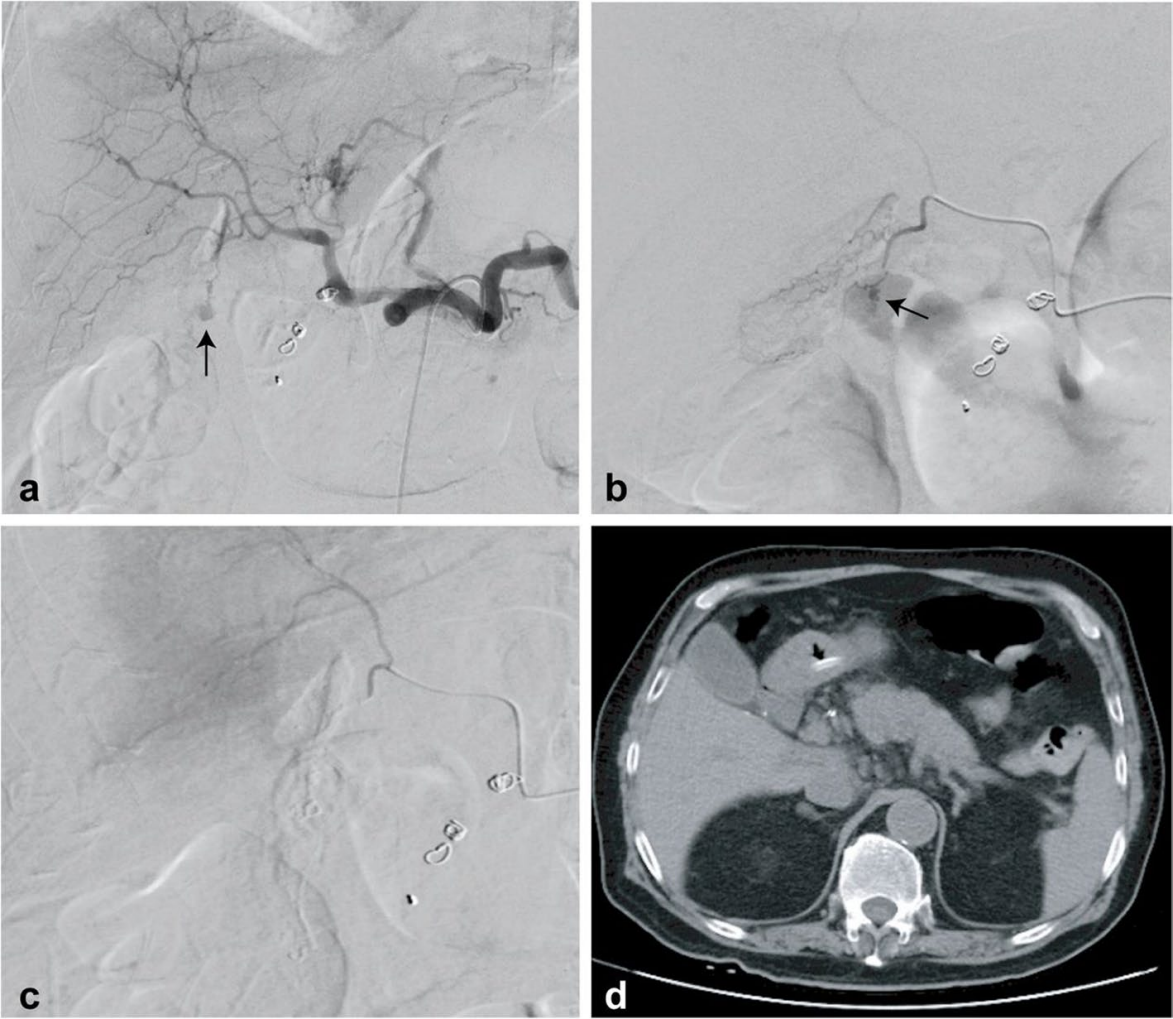
Case 2. **a** Angiography after empirical gastroduodenal artery embolization shows contrast extravasation (arrow). **b** Super-selective angiography confirms the source of bleeding is the cystic artery (arrow). **c** The cystic artery is embolized with a mixture of NBCA and iodized oil in a 1:4 ratio. **d** One month post-procedural CT scan shows no gallbladder infraction

An 81-year-old male was admitted to the hospital with erysipelas of his left lower limb. The patient had a history of gouty arthritis and was taking non-steroidal anti-inflammatory drugs (NSAIDs). He had no previous history of biliary system diseases. One week after admission, the patient presented with hematemesis and shock. An urgent endoscopic examination revealed a post-bulbar ulcer with active bleeding. Despite endoscopic and pharmacological treatments, the patient’s hemodynamic stability was compromised. Celiac angiography was performed but failed to identify the source of bleeding. Consequently, an empirical embolization of the gastroduodenal artery using coils and gelatin sponge particles was performed. After embolization, a follow-up angiogram demonstrated contrast extravasation (Fig. 2a), and super-selective angiography confirmed the source as the cystic artery (Fig. 2b). The cystic artery was embolized with N-butyl-cyanoacrylate (NBCA) mixed with iodized oil at a ratio of 1:4 (Fig. 2c). After the embolization, the patient’s circulation stabilized. Post-embolization CT imaging revealed minimal intraperitoneal free air, suggestive of a potential duodenal perforation. A nasojejunal tube was inserted for enteral feeding. At one-month follow-up, a CT scan showed no signs of gallbladder infraction or pneumoperitoneum (Fig. 2d). Endoscopic examination confirmed healing of the ulcer, and the nasojejunal tube was subsequently removed.

### Case3

**Fig. 3:**
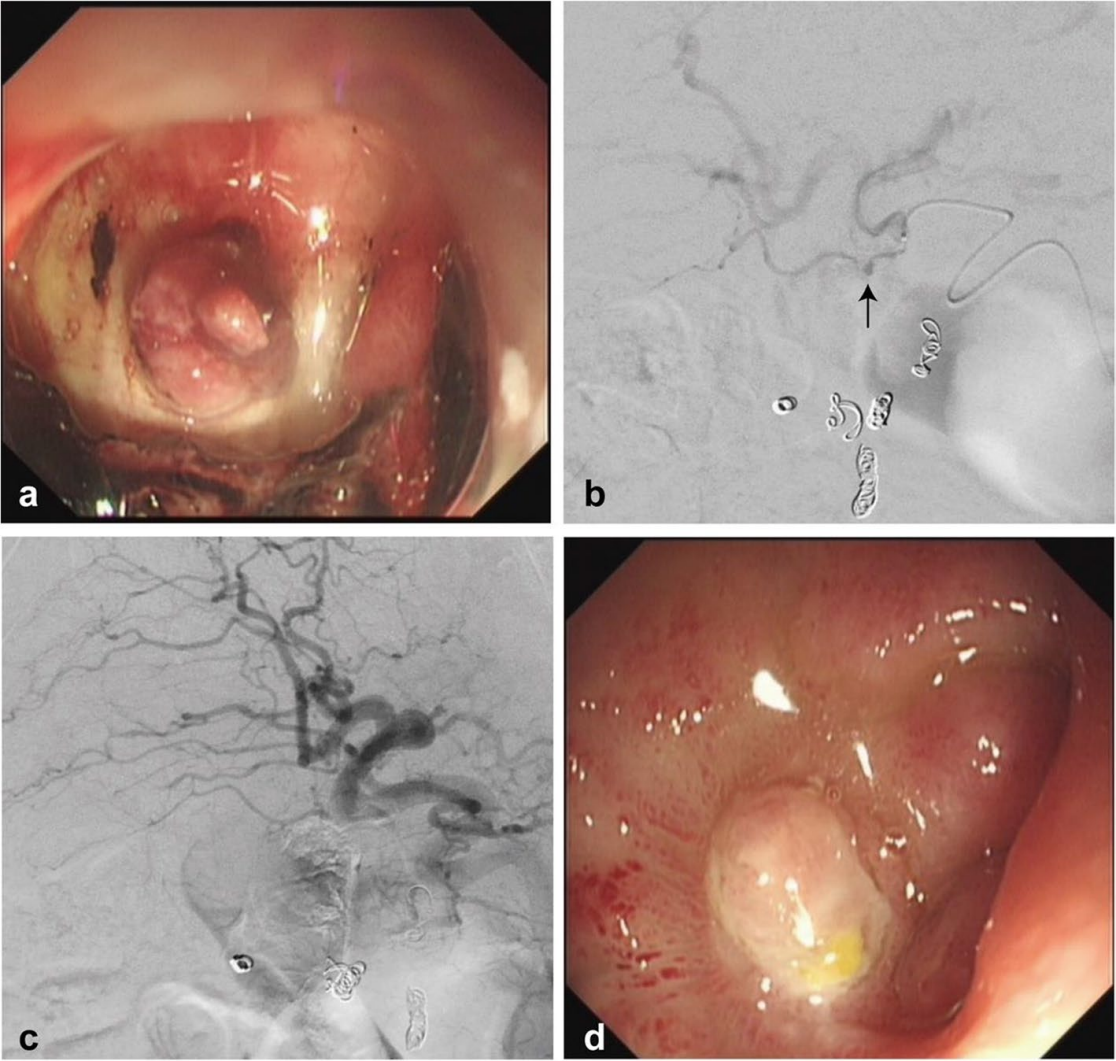
Case3. **a** Endoscopic examination shows an anterior duodenal ulcer with exposed vessels and scab attachment. **b** Super-selective catheterization of the cystic artery reveals a pseudoaneurysm (arrow). **c **Embolization of the cystic artery is performed using a 1:4 mixture of NBCA and iodized oil. **d** Follow-up endoscopy ten days after cystic artery embolization shows complete healing of the ulcer

An 82-year-old male patient was admitted with hematemesis. The patient had a history of osteoarthritis treated with NSAIDs and denied any prior biliary diseases. An emergent endoscopic examination revealed an anterior-bulb duodenal ulcer that did not respond to endoscopic treatment (Fig. 3a). Subsequently, an emergency angiography was performed, but no positive findings were observed. Empirical gastroduodenal artery embolization was performed using gelatin sponge particles and coils, but rebleeding occurred, therefore, a repeat angiography was conducted. A pseudoaneurysm of the cystic artery was identified following super-selective catheterization of the cystic artery (Fig. 3b). The pseudoaneurysm was successfully embolized with NBCA (Fig. 3c). Ten days after the embolization, an endoscopic examination revealed that the ulcer had healed (Fig. 3d). The patient was discharged without gallbladder infraction.

## Discussion

Duodenal ulcers that erode into the cystic artery are a rare cause of UGIB. The literature describes a limited number of cases, with three located in the anterior bulb, four in the post-bulbar region, and the site was unknown for six cases [1–9]. In this study, we report three additional cases that confirm the predominance of cystic artery erosion occurring in the anterior-bulb or post-bulbar regions.

Transcatheter arterial embolization (TAE) is considered the first-line treatment for UGIB that is refractory to medical and endoscopic therapy [10]. However, a significant challenge in TAE is the occurrence of negative angiography findings, and empirical embolization is recommended in this scenario [11, 12]. In our three patients, initial celiac angiography did not reveal positive findings. In the first patient, super-selective angiography of the cystic artery was performed based on CT findings of contrast agent extravasation near the gallbladder, resulting in a positive result. In the second patient, contrast agent extravasation was observed during celiac angiography after empirical embolization of the gastroduodenal artery, and super-selective angiography confirmed bleeding from the cystic artery. The third patient experienced rebleeding after empirical embolization, prompting super-selective angiography of the cystic artery based on the experiences from the previous two cases and previous literature reports, which resulted in a positive finding. These cases suggest that super-selective angiography of the cystic artery should be considered for patients with UGIB due to ulcer in the anterior-bulb or post-bulbar regions who do not respond to empirical embolization.

TAE offers a less invasive alternative to surgery, making it particularly beneficial for high-risk patients. Historical concerns about gallbladder infarction have favored surgical intervention over direct cystic artery embolization. However, non-target embolization of the cystic artery during trans-arterial chemoembolization is not uncommon but is typically asymptomatic and self-limiting [13]. In the study by Hyo-Cheol et al. [9] among the 20 patients who underwent TAE for cystic artery bleeding, ischemic cholecystitis developed in three cases. Notably, three patients whose bleeding was attributed to duodenal ulcer erosion did not experience ischemic cholecystitis following the TAE procedure. In our three patients, no gallbladder infarction was observed regardless of the embolization material used. There are collateral vessels between the hepatic capsule arteries and the cystic artery [14], which likely prevented the gallbladder from infarction after the embolization of the cystic artery.

In conclusion, ulceration eroding into the cystic artery is a rare etiology of UGIB, predominantly associated with anterior or post-bulbar duodenal ulcers. Our case series demonstrate the safety and efficacy of super-selective embolization of the cystic artery as a viable treatment option in such scenarios. Future studies with larger sample sizes are necessary to further validate these findings.

## Data Availability

Data sharing is not applicable to this article as no datasets were generated or analysed during the current study.

## Abbreviations

UGIB: Non-variceal upper gastrointestinal bleeding
CT: Computed tomography
NSAIDs: Non-steroidal anti-inflammatory drugs
NBCA: N-butyl-cyanoacrylate
TAE: Transcatheter arterial embolization
